# Phytochemical property and assessment of antidermatophytic activity of some selected wild macrofungi against pathogenic dermatophytes

**DOI:** 10.1080/21501203.2016.1145608

**Published:** 2016-02-22

**Authors:** Olusola Clement Ogidi, Victor Olusegun Oyetayo

**Affiliations:** Department of Microbiology, Federal University of Technology, PMB 704, Akure, Nigeria

**Keywords:** Dermatophytes, wild mushrooms, phytochemicals, antifungal agents

## Abstract

The phytochemical property and inhibitory potential of petroleum ether and ethanol extracts of *Lenzites quercina, Ganoderma lucidum* and *Rigidoporus ulmarius* were assessed. Standard method was adopted to quantify the phytochemicals in the mushroom extracts. Poisoned food technique was used to assess the inhibition of the extracts obtained from the macrofungi against some pathogenic dermatophytes. The phenolic content of the studied mushroom extracts ranged from 15.7 to 83.8 mg gallic acid equivalent (GAE)/g extract. Ethanolic extract of *G. lucidum* has the higher content of alkaloids (34.3 mg/g) and flavonoids (18.1 mg/g). Exactly 33.1 mg/g of terpenoids and 16.6 mg/g of saponins were also quantified in ethanolic extract of *L. quercina*. Extracts of *L. quercina, G. lucidum* and *R. ulmarius* exhibited wide range of mycelia inhibition at 50 mg/ml on the tested dermatophytes. The consistent inhibition displayed by the mushroom extracts against the dermatophytes affirms that these wild mushrooms contain bioactive compounds that are antifungal in nature and may possess the ability to cure dermatophyte infections.

## Introduction

Dermatophyte infections are common diseases of cutaneous and subcutaneous part of human and animal body. Dermatophytes are pathogenic fungi that have the ability to colonize, degrade and paralyse the keratin membrane of the skin due to the presence of proteolytic enzymes, which it uses to hydrolyze keratin, the main protein constituent of hair, nails, feet and skin (Weitzman and Summerbell 1995). Dermatophyte infections are commonly spread by direct contact with infected person, animals, soil or indirect contact with formites (Prescott et al. 2004).

There are cases of infection caused by dermatophytes, which are difficult to treat due to low response of patient to some antifungal drugs, discontinuous medication, inappropriate self-medication and failure to comply with clinical guideline of the drug (Del Palacio et al. 2000). The conditions listed above often result to relapse of the infections. Hence, this has been a significant health problem and thus requires prompt attention.

The current epidemiological data had shown increasing incidence of fungal infection among the school age pupils and immunocompromised patients, in whom it has been a life-threatening issue (Ndako et al. 2012). The resistance patterns of dermatophytes to several and commonly used antifungal drugs during the treatment had compounded the spreading of dermatophyte infections. Besides the prevalence of resistance of dermatophytes, many synthetic antifungal drugs had also been associated with some adverse side effects such as hepatotoxicity, nausea, diarrhoea and impotency (Del Palacio et al. 2000). This occurs as result of cellular similarities between the fungi and mammalian, which make the treatment of dermatophyte infections to be tedious, expensive and time consuming (Soares et al. 2013). Hence, there is a need to provide affordable and appropriate treatment for dermatophyte infections without side effects.

Mushrooms are known to possess medicinal properties. In the last two decades, the health-promoting effect of mushrooms had gained more attention due to the large array of secondary metabolites present in the fruit bodies and submerged culture. The potential of wild mushrooms as a source of bioactive compounds has prevented the manifestation of some illness (Alves et al. 2013). The medicinal importance of mushroom requires a continuous searching and exploiting wild macrofungi for antimicrobial, antioxidants, anticancer and anti-inflammatory. It is therefore worthwhile to screen extracts of *Lenzites quercina, Ganoderma lucidum* and *Rigidoporus ulmarius* against some pathogenic dermatophytes.

## Materials and methods

### Source of dermatophytes and identification

The pathogenic dermatophytes used in this study were sourced from General Hospital, Ikare-Akoko, Ondo State, Nigeria. The dermatophytes were isolated from school pupil and immune compromised patients from August to November, 2013. The viability and identity of the dermatophytes were confirmed by culturing onto solidified Sabouraud dextrose agar (SDA) with chloramphenicol (50 mg/L) and cycloheximide (500 mg/L). The plates were incubated at room temperature (28 ±1°C) and observed for growth. Macroscopic and microscopic examinations were interpreted according to Frey et al. (1979) to ascertain the identity of dermatophytes to species level.

### Studied wild macrofungi and preparation of their extracts

*L. quercina* and *G. lucidum* were collected from a farmland near Aule Street in September, 2013, while *R. ulmarius* was sourced from another farmland in Obele Estate, Akure, in November, 2013. These farmlands are (1.0–1.1) km to the Federal University of Technology, Akure, Nigeria (Lat 07° 14^I^N Long 05° 11^I^E). The mushrooms were identified morphologically and confirmed by molecular tools using the internal transcribed spacer region of the rDNA. The mushrooms were dried at 35 ±2°C in oven (DHG9053-A) for 6 days. Dried sample of *L. quercina, G. lucidum* and *R. ulmarius* were ground into powder by mill machine (Retsch GmbH 5657 HAAN). Milled sample of each mushroom (100 g) was soaked in 1000 ml of petroleum ether (95 % v/v) and ethanol (95 % v/v) for 72 h. The filtrates obtained through filter paper Whatman no. 1 were concentrated in rotary evaporator (RE-52A, UNION Laboratories, England) and the dried extracts were designated as PLQ (petroleum ether extract of *L. quercina*), ELQ (ethanol extract of *L. quercina*), PGL (petroleum ether extract of *G. lucidum*), EGL (ethanol extract of *G. lucidum*), PRU (petroleum ether extract of *R. ulmarius*) and ERU (ethanol extract of *R. ulmarius*).

### Phytochemical screening of the mushroom extracts

The mushroom extracts were screened qualitatively and quantitatively for alkaloids, terpenoids, sterols, tannins, saponins, phlobataninns, anthraquinones and cardic glycosides using standard methods of Sofowora (1993), Harborne and Baxter (1995) and Trease and Evans (2005).

### Determination of total phenol contents

The total phenolic content of extracts obtained from wild macrofungi was determined according to the method of Singleton et al. (1999). Briefly, aliquot of the extracts (0.5 ml) were taken in a 10 ml glass tube and 2.5 ml of 10% Folin–Ciocalteu reagent was added. After 2 min, 2 ml of 7.5% sodium carbonate was added. The mixed solution was incubated for 1 h at 25°C. Thereafter, the absorbance was measured at 765 nm and compared to a gallic acid calibration curve. Total phenols were determined as gallic acid equivalents (mg GAE/g extract) in triplicate.

### Determination of flavonoid contents

The total flavonoid content of the studied mushroom extracts was determined using a method reported by Meda et al. (2005). Briefly, diluted sample (0.5 ml) of extract was mixed with 0.5 ml methanol, 100 µl of 10% aluminium trichloride (AlCl_3_), 50 µl of potassium acetate (1.0 M) and 1.5 ml water was mixed and incubated at room temperature for 30 min. The absorbance of the reaction was subsequently measured at 415 nm. The total flavonoid content was calculated as:

$$Conc.\;of\;sample=\frac{Abs\;of\;sample\times conc.\;of\;Std}{Abs\;of\;Std}$$

where Abs = absorbance, Std = standard.

### Determination of antifungal activity of the mushroom extracts

The antifungal activities of mushroom extracts were determined by poisoned food technique (Parajuli et al. 2005). Briefly, the concentration of mushroom extracts was varied from 10 mg/ml to 50 mg/ml when reconstituted in 20% v/v of dimethyl sulphoxide (DMSO). Aliquot of 1.0 ml of each concentration was aseptically poured into Petri dish followed by adding sterilized SDA. The Petri dish was shaken while adding SDA, so as to get even mixture of the contents. Seven days old culture of the fungi was used to prepare inoculum (6.0 mm discs) using cork borer. A single disc was aseptically placed upside down in the centre of each plate containing medium and extract. The average diameter of fungal colonies was measured on the 7th day after inoculation and percentage of mycelia growth inhibition was calculated. The control set contained only DMSO as a negative control and griseofulvin and fluconazole as positive control. The test was carried out in replicates and percentage of mycelia inhibitions at different concentrations was calculated using 

$$\frac{g_{c}-g_{t}}{g_{c}}\times 100$$

where *g_c_* = growth of mycelia colony after incubation period in control set subtracting the diameter of inoculums disc, and *g_t_* = growth of mycelia colony after incubation period in treatment set subtracting the diameter of inoculums disc.

## Results

Table 1 shows the phytochemical contents of the mushroom extracts obtained from pet-ether and ethanol. The phenol contents of the mushroom extracts ranged from 15.7 to 83.8 mg GAE/g extract. Ethanolic extract of *L. quercina* had the highest content of saponins (16.6 mg/g) and terpenoids (33.1 mg/g). This amount (21.7 mg/g) of steroids was quantified in petroleum ether extract of *G. lucidum* while 34.3 mg/g of alkaloids and18.1 mg/g of flavonoids were also found in ethanolic extract of *G. lucidum*. The quantities of phlobatannins and anthraquinones were low in the examined mushroom extracts with value ranging from 0.010 to 0.014 mg/g. Cardiac glycosides have values of (0.16–26.6) mg/g for the mushroom extracts.

**Table 1.** Phytochemical contents (mg/g) in wild mushroom extracts of petroleum ether and ethanol.

phytochemicals	PLQ	ELQ	PGL	EGL	PRU	ERU
Alkaloids	3.1^e^ ± 0.0	19.2^b^ ± 0.0	2.0^f^ ± 0.3	34.3^a^ ± 0.0	8.4^d^ ± 0.0	12.9^c^ ± 0.1
Saponins	16.6^a^ ± 0.10	7.9^b^ ± 0.2	0.78^e^ ± 0.0	1.1^d^ ± 0.0	0.81^e^ ± 0.0	4.11^c^ ± 0.0
Tannins	2.0^b^ ± 0.02	4.0^a^ ± 0.0	0.00	3.8^a^ ± 0.0	0.00	0.8^c^ ± 0.0
Terpenoids	9.5^d^ ± 0.40	33.1^a^ ± 0.0	15.5^c^ ± 0.0	23.9^b^ ± 0.01	0.66^e^ ± 0.0	14.83^c^ ± 0.0
Steroids	5.8^d^ ± 0.30	10.9^b^ ± 0.02	21.7^a^ ± 0.2	0.80^e^ ± 0.0	11.46^b^ ± 0.2	7.54^c^ ± 0.0
Phlobatanins	0.00	0.00	0.012^a^ ± 0.0	0.012^a^ ± 0.0	0.00	0.010^b^ ± 0.0
Flavonoids	9.11^e^ ± 0.0	11.10^d^ ± 0.0	13.0^bc^ ± 0.21	18.1^a^ ± 0.03	8.80^e^ ± 0.02	13.1^bc^ ± 0.0
Anthraquinones	0.00	0.00	0.00	0.014 ± 0.00	0.00	0.00
Cardic glycosides	5.4^e^ ± 0.02	13.2^b^ ± 0.04	10.2^c^ ± 0.0	26.6^a^ ± 0.01	0.16^e^ ± 0.05	5.18^d^ ± 0.01
Phenol*	35.0^d^ ± 1.0	71.6^ab^ ± 0.02	30.8^de^ ± 0.33	83.8^a^ ± 0.11	15.7^f^ ± 0.5	48.8^c^ ± 0.15

Notes: PLQ: petroleum ether extract of *Lenzites quercina*, ELQ: ethanol extract of *Lenzites quercina*, PGL: petroleum ether extract of *Ganoderma lucidum*, EGL: ethanol extract of *Ganoderma lucidum*, PRU: petroleum ether extract of *Rigidoporus ulmarius* and ERU: ethanol extract of *Rigidoporus ulmarius*. Values are mean ± SD of replicates (*n* = 3). Values with the same alphabet are not significantly different at (*P* = 0.05). *Gallic acid equivalent (mg GAE/g extract).

The inhibitory potentials of the mushroom extracts at different concentrations are shown in Tables 2^Table 3.^–4. The mycelia inhibition was dose-dependent from (10 to 50) mg/ml of the mushroom extracts. Some species of dermatophytes such as *Epidermatophyton floccosum, Trichophyton yaoundei, Microsporum ferrugineum, T. tonsurans, M. nanum* and *T. verrucosum* were not susceptible to the mushroom extracts obtained from petroleum ether at 10 mg/ml (Table 2). The mycelia inhibitory effect observed at the 10 mg/ml was lower compared to what was obtained at 20 mg/ml and 50 mg/ml (Tables 2–4). At the highest concentration of 50 mg/ml, the mushroom extracts exhibited wide range of mycelia inhibition against tested dermatophytes. *L. quercina* extracts possess mycelia inhibition of 25.5–78.9% while *G. lucidum* extract had 28.8–85.5% and 20.4–74.4% was observed for *R. ulmarius* extract (Table 4). Griseofulvin and fluconazole, the positive controls and the extracts have similar inhibitory activities on some dermatophytes as shown in Table 4. Mushroom extracts effectively inhibited all the dermatophytes at the concentration of 50 mg/ml.

**Table 2.** Percentage of mycelia inhibition by mushroom extracts and commercial antifungals against tested dermatophytes at 10 mg/ml.

Dermatophytes	PLQ	ELQ	PGL	EGL	PRU	ERU	GRE	FLU
*Epidermatophyton floccosum*	–	–	–	10.6 ± 0.0	–	–	18.0 ± 0.0	–
*Trichophyton yaoundei*	–	6.0 ± 0.0	–	8.0 ± 0.0	–	–	24.0 ± 0.0	12.0 ± 0.3
*Microsporum ferrugineum*	–	–	–	–	–	–	48 ± 1.0	–
*T. tonsurans*	–	28.9 ± 0.8	–	29.7 ± 0.17	–	21.8 ± 0.0	–	23.0 ± 0.3
*M. nanum*	–	–	–	–	–	–	30.0 ± 0.1	–
*T. mentagrophytes*	–	–	28.3 ± 0.0	21.0 ± 0.9	23.0 ± 0.5	–	36.0 ± 0.0	–
*M. gypseum*	10.3 ± 0.01	33.1 ± 1.2	–	–	–	–	–	40.0 ± 2.1
*T. verrucosum*	–	–	–	16.0 ± 0.0	–	10.3 ± 0.0	–	24.0 ± 2.2
*T. rubrum*	29.1 ± 0.3	10.0 ± 0.0	–	–	–	–	18.8 ± 0.7	10.1 ± 0.0

PLQ: petroleum ether extract of *Lenzites quercina*, ELQ: ethanol extract of *Lenzites quercina*, PGL: petroleum ether extract of *Ganoderma lucidum*, EGL: ethanol extract of *Ganoderma lucidum*, PRU: petroleum ether extract of *Rigidoporus ulmarius* and ERU: ethanol extract of *Rigidoporus ulmarius*, GRU: griseofulvin, FLU: fluconazole and – = no mycelia inhibition. Values are mean ± SD of replicates (*n* = 3).

**Table 3.** Percentage of mycelia inhibition by mushroom extracts and commercial antifungals against tested dermatophytes at 20 mg/ml.

Dermatophytes	PLQ	ELQ	PGL	EGL	PRU	ERU	GRE	FLU
*Epidermatophyton floccosum*	–	24.1 ± 1.3	11.3 ± 0.3	40.1 ± 0.02	–	29.2 ± 0.0	42.0 ± 0.02	–
*T. yaoundei*	–	18.6 ± 0.2	–	23.4 ± 0.1	27.0 ± 0.3	–	60.0 ± .00	23.0 ± 0.0
*M. ferrugineum*	–	–	–	16.4 ± 0.04	–	35.0 ± 0.0	61.0 ± 0.0	–
*T. tonsurans*	33.1 ± 0.4	49.7 ± 2.0	–	61.9 ± 0.05	–	–	–	48.3 ± 0.1
*M. nanum*		20.3 ± 0.8	20.1 ± 0.0	28.3 ± 0.5	–	–	55.0 ± 0.12	–
*T. mentagrophytes*	18.3 ± 0.6	–	36.3 ± 0.0	48.4 ± 0.4	23.0 ± 0.02	33.0 ± 0.8	53.0 ± 0.25	13.7 ± 0.3
*M. gypseum*	29.0 ± 0.1	41.8 ± 0.3	–	38.1 ± 0.0	47.0 ± 0.2	–	–	48.0 ± 0.3
*T. verrucosum*	–	–	–	–	31.5 ± 0.0	50.3 ± 1.3	37.0 ± 0.9	45.0 ± 0.2
*T. rubrum*	36.1 ± 0.0	28.6 ± 0.0	29.0 ± 0.1	–	–	–	38.0 ± 0.6	30.2 ± 0.0

PLQ: petroleum ether extract of *Lenzites quercina*, ELQ: ethanol extract of *Lenzites quercina*, PGL: petroleum ether extract of *Ganoderma lucidum*, EGL: ethanol extract of *Ganoderma lucidum*, PRU: petroleum ether extract of *Rigidoporus ulmarius* and ERU: ethanol extract of *Rigidoporus ulmarius*, GRU: griseofulvin, FLU: fluconazole and – = no mycelia inhibition. Values are mean ± SD of replicates (*n* = 3).

**Table 4.** Percentage of mycelia inhibition by mushroom extracts and commercial antifungals against tested dermatophytes at 50 mg/ml.

Dermatophytes	PLQ	ELQ	PGL	EGL	PRU	ERU	GRE	FLU
*Epidermatophyton floccosum*	–	68.9 ± 0.13	55.1 ± 0.2	79.1 ± 0.0	–	64.7 ± 0.3	68.8 ± 0.0	24.1 ± 0.0
*T. yaoundei*	–	44.6 ± 0.2	–	58.2 ± 0.0	50 ± 0.23	53 ± 0.51	83.1 ± 1.1	56.3 ± 0.0
*Microsporum ferrugineum*	38.4 ± 0.5	26.4 ± 1.0	–	29.3 ± 0.0	–	72.5 ± 0.32	79.0 ± 0.0	28.8 ± 0.5
*T. tonsurans*	54.0 ± 0.0	78.9 ± 0.4	33.1 ± 0.3	82.7 ± 1.2	–	–	23 ± 0.0	79.1 ± 0.0
*M. nanum*	–	30.5 ± 0.0	56 ± 0.21	60.3 ± 0.0	–	–	94.4 ± 0.0	28.0 ± 0.0
*T. mentagrophytes*	38.5 ± 0.0	33.1 ± 0.3	73.1 ± 0.0	85.5 ± 1.5	58.5 ± 0.20	68.3 ± 0.0	75.2 ± 0.0	37.7 ± 0.3
*M. gypseum*	60.3 ± 0.2	78.3 ± 1.1	–	71.8 ± 0.8	64.4 ± 0.2	–	-	83.3 ± 1.4
*T. verrucosum*	–	–	–	38.0 ± 0.0	51.4 ± 0.0	74.4 ± 0.0	62.1 ± 1.3	74.7 ± 0.5
*T. rubrum*	59.6 ± 0.0	25.5 ± 0.0	57.3 ± 0.12	28.8 ± 0.5	–	20.4 ± 0.5	60.4 ± 1.1	74.6 ± 0.5

PLQ: petroleum ether extract of *Lenzites quercina*, ELQ: ethanol extract of *Lenzites quercina*, PGL: petroleum ether extract of *Ganoderma lucidum*, EGL: ethanol extract of *Ganoderma lucidum*, PRU: petroleum ether extract of *Rigidoporus ulmarius* and ERU: ethanol extract of *Rigidoporus ulmarius*, GRU: griseofulvin, FLU: fluconazole and – = no mycelia inhibition. Values are mean ± SD of replicates (*n* = 3).

## Discussion

The cost of convectional antifungal drugs coupled with persistent resistance of fungi and the long-term treatment of dermatophyte infections requires alternative treatments that will be safe and reliable. The use of ethnoparmacological agents from medicinal mushrooms for the treatment of dermatophyte infections was investigated. This study was therefore undertaken to assess the inhibitory potential of extracts obtained from *L. quercina, G. lucidum* and *R. ulmarius* against some pathogenic dermatophytes. Phytochemical constituents found in studied mushroom extracts are phenol, flavonoids, alkaloids, steroids, saponins, terpenoids and cardiac glycosides. Findings of Unekwu et al. (2014) had reported most of these phytochemicals as functional ingredients in a number of medicinal mushrooms. These phytochemicals are known to function as antibacterial, antifungal, antioxidants and anticancer agents (Cowan 1999; Sokovic et al. 2013). Thus, phytochemicals in medicinal mushrooms had tremendous potentials on the health care system by preventing several degenerative diseases and physiological disorders. These significant impacts have accorded medicinal mushrooms as nutraceutical agent.

Appreciable quantity of phenol, alkaloids, flavonoids, terpenoids, steroids, taninns and cardiac glycosides were found in the extracts of *L. quercina, G. lucidum* and *R. ulmarius*. De Silva et al. (2013) had reported that wild mushrooms harbours wealthy quantity of valued bioactive compounds. Hence, the adequate exploitation of these biologically active compounds in wild macrofungi could bring end to the perpetual failure witnessing in some chemotherapies.

Extracts of *L. quercina, G. lucidum* and *R. ulmarius* exhibited wide range of mycelia inhibition against dermatophytes like the commonly used antifungal agents (positive control). The mycelia inhibition caused by mushroom extracts against the dermatophytes is similar to report of Devi et al. (2013), Sagar and Vidyasagar (2013) and Shinkafi (2013) who had tested various medicinal plants against dermatophytes. This shows that medicinal product from plants and mushrooms could be continually sourced and adequately utilized to treat dermatophyte infections. Kim et al. (2008) had reported the inhibitory effect of *Clitocybe nebularis* on *Trichophyton mentagrophytes* with inhibition zones of (9–11) mm. The inhibitory effect displayed by the mushroom extracts is a reflection of the diversities of pharmacological agents in mushrooms.

Wong et al. (2010) had attributed the antifungal activity of wild mushrooms to the presence of glucanase, which exhibits antifungal activity by hydrolyzing glycan in fungal wall and thereby producing a weakened cell wall and lysed the cell. The antifungal activities of *G. lucidum* had been associated with the presence of ganoduric protein (Wang and Ng 2006). Ogidi et al. (2015) associated the antimicrobial activity of *L. quercina* to the presence of fatty acids and other phytochemicals. The ability of phytochemicals in the mushroom extracts to penetrate cellular membranes, cell walls and cytoplasmic constituents of pathogenic fungi could lead to the inhibition of these human infectious fungal pathogens. This is in line with Tiwari et al. (2009) who had suggested that most of the phytochemicals have the ability to interact with the plasma membrane of microorganisms and alter its permeability, and eventually lead to leakage of ions. The inhibitory property of the mushroom extracts against pathogenic dermatophytes in the present study shows that these mushrooms could be a promising source of antidermatophytic agent. The need for further exploration of bioactive compounds in medicinal mushroom as an alternative to commercial antifungal is therefore expedient.

## Conclusion

Commercial antifungal agents are bedeviled with the following shortcomings: drugs interaction, several toxicities associated with long-term use of antifungal drugs and the resistance displayed by the pathogenic fungi. Extract from mushrooms which contain some secondary metabolites displayed promising antifungal property against dermatophytes may therefore be used as effective alternative. Further study aimed at formulating antifungal cream from wild macrofungi for the treatment of dermatophyte infections is the next stage of our study.
