# Correction to: Ogidi and Oyetayo, Phytochemical property and assessment of antidermatophytic activity of some selected wild macrofungi against pathogenic dermatophytes

**DOI:** 10.1080/21501203.2016.1168079

**Published:** 2016-04-04

Ogidi OC and Oyetayo VO. 2016. Phytochemical property and assessment of antidermatophytic activity of some selected wild macrofungi against pathogenic dermatophytes. *Mycology*. http://dx.doi.org/10.1080/21501203.2016.1145608

When the above article was first published online, the value given in Table 3, column 5, row 7 was wrongly given as “38.8.1”. This has now been corrected to “38.1” in both the print and online versions.

